# Effects of Thermochromic Fluorane Microcapsules and Self-Repairing Waterborne Acrylic Microcapsules on the Properties of Water-Based Coatings on Basswood Surface

**DOI:** 10.3390/polym14122500

**Published:** 2022-06-19

**Authors:** Pan Pan, Xiaoxing Yan, Lin Wang

**Affiliations:** 1 Co-Innovation Center of Efficient Processing and Utilization of Forest Resources, Nanjing Forestry University, Nanjing 210037, China; pan@njfu.edu.cn; 2 College of Furnishings and Industrial Design, Nanjing Forestry University, Nanjing 210037, China; wanglin@njfu.edu.cn

**Keywords:** film properties, microcapsules, self repair, thermochromism

## Abstract

The effect of the addition of fluorane microcapsules and urea formaldehyde resin (UF) waterborne acrylic resin microcapsules on the comprehensive properties of the water film on the surface of basswood was studied. Three-factor and two-level orthogonal experiments were carried out with “fluorane microcapsule content”, “aqueous acrylic resin microcapsule content” and the “fluorane microcapsule addition method” to prepare a self-repairing thermochromic coating. The optical, mechanical, microstructure and self-repairing properties of the film were optimized by independent experiments on the maximum influence factors of the fluorane microcapsule content. It was concluded that the topcoat with 15% fluorane microcapsules and primer added with 15% water acrylic resin microcapsules had better comprehensive properties. The temperature range was 30–32 °C, the color difference at 32 °C was 72.6 ± 2.0, the 60° gloss was 3.3%, the adhesion was 0 grade, the hardness was 4 H, the impact resistance was 15.0 ± 0.8 kg∙cm, the elongation at break was 17.2% and the gap width was reduced by 3.5 ± 0.1 μm after the film was repaired. The repair rate reached 62.5%. By using microcapsule embedding technology, the repair agent and discoloration agent are embedded in the matrix. The waterborne acrylic resin microcapsules can effectively inhibit crack formation in the coating, and the fluorane microcapsules can achieve the thermochromic property of the coating. This study provides a new research idea for the self-repairing thermochromic dual function of a water-based coating.

## 1. Introduction

As a natural polymer heterogeneous composite material [1], wood has always been favored by people and has always maintained an important position in the field of building decoration materials [2]. However, due to changes in environmental factors [3], wood’s wet expansion and dry shrinkage [4], insufficient film toughness [5] and mismatches with the wood’s interface [6], cracks appear in the film during use [7,8], which reduces the sealing performance and leads to film failure. Microcapsule technology is widely used in coatings, wood products, furniture, agriculture and so on. Yuan [9] used melamine formaldehyde (MF) resin microcapsules to improve the efficacy of pesticides and the influence of process conditions on the properties of microcapsules [10]. Uzoma et al. [11] stated that the multi-stimulus response coating prepared by UF has the effect of corrosion protection. Fang et al. [12] studied improvements in the interfacial adhesion with wood veneer, but this may damage wood at the same time. Therefore, in the application of microcapsule technology, the response between the microcapsule and substrate should be fully considered, and new functions can be given without changing the properties of the wood substrate. The self-repairing coating prepared by microcapsule technology [13] can effectively solve the generation of microcracks. Cho et al. [14] were able to automatically repair and prevent the corrosion of the underlying substrate by dispersing microencapsulated healing agents in the polymer film to form a self-repairing coating. Samadzadeh et al. [15] added microcapsules into the coating and quickly released the repair agent after the crack in the coating expanded, so that the coating had the ability of self-repairing. The waterborne acrylic resin coating [16] takes water as the dispersion medium, which has excellent mechanical properties, a good compatibility mode and easy modification. It has the characteristics of green environmental protection, is safe and has high efficiency. It is suitable to be used as the capsule core of microcapsules, which can effectively heal the generation of microcracks and achieve the role of being self repairing. Zhu et al. [17] reported a UV responsive, bifunctional microsphere system for repairing coating cracks for efficient self-healing coatings. Therefore, the self repair of a water-based coating on a wood surface can further protect the wood substrate.

With the improvement in people’s taste, there are greater requirements for the intelligent [18,19] and visual use of decorative materials. The application of thermochromic coatings to wood surfaces can not only provide people with excellent visual effects [20], but also meet users’ personalized needs for decorative materials. Therefore, it has been broadly developed in decoration and other fields. In addition, wood often needs surface treatment before use, and its discoloration characteristics mainly depend on the material’s surface, while fluorane dyes themselves are colorless or a light color, which can produce different colors through different types and structures of substituents [21]. Because of its small particle size, thin wall thickness and large specific surface area, microcapsule technology can guarantee the heat transfer area and increase the heat transfer efficiency [22,23], thus effectively improving the thermochromic properties. The fluorane microcapsules can effectively realize the thermochromism of wood surfaces. Liu et al. [24] modified the background color and material color of poplar to obtain the best value. Yan et al. [25] studied the changes in the coating films of color-changing microcapsules in different seasons and temperatures. Zhu et al. [26] prepared transparent thermochromic microcapsules by an in situ method, which can be used in intelligent wood coatings. The application of color-changing microcapsules to wood coating is a highly effective method. It is worth studying to endow the coating with new functions. Jamil et al. [27] studied a clean and smooth photothermal coating that shows excellent deicing performance for a long time at low temperatures. Aziz et al. [28] studied nanocomposites that were evenly dispersed in bio-based epoxy resin to obtain bonding properties. Ahmed [29] reviewed the technical applications of nanocomposites with different functional materials and geometric shapes. At present, there are few reports about the self healing and discoloration of water-based coatings on wood surfaces at the same time. The water-based coating takes water as the dispersion medium, which is environmentally friendly and green. The texture of the water-based coating is thin, which can better adapt to the material’s surface [30]. Therefore, two kinds of microcapsules, thermochromic and self healing, are added to the water-based coating, respectively, so that the film can realize the dual functions of being self repairing and thermochromic.

Therefore, in this paper, to prepare the water-based coating on the surface of the basswood with excellent thermochromic and self-healing functions, the addition method and preparation process of color change and self-healing microcapsules applied to the primer and topcoat were studied. The effects of the independent addition of thermochromic microcapsules (fluorane microcapsules) and UF @ waterborne acrylic acid microcapsules on the comprehensive properties of the water-based coating on the surface of basswood were discussed. The UF @ waterborne acrylic acid-coated microcapsules (hereinafter referred to as waterborne acrylic resin microcapsules) were compounded by the method of in situ polymerization [31,32,33]. The three factors of “fluorane microcapsule content”, “water-based acrylic resin microcapsule content” and the “fluorane microcapsule addition method” were selected for orthogonal experiments. The prepared film sample had the better thermochromic performance, so the biggest influencing factor on the film’s discoloration performance is obtained. Then the single factor independent experiment was conducted out to find the most influential factors. Combined with the results of the orthogonal horizontal experiment, the effects of waterborne acrylic and fluorane microcapsules on the properties of the water-based coating on basswood’s surface were explored through the analysis of its optical and mechanical self-healing properties and microstructure. The best preparation process for the water-based coating on basswood’s surface was determined to realize the self-repairing and thermochromic coating, so as to broaden the use scope and application prospect of wood and create more social benefits.

## 2. Materials and Methods

### 2.1. Experimental Materials

The reagents required for synthesizing UF @ waterborne acrylic acid microcapsules and testing the coating rate of microcapsules are shown in Table 1. The 1,2-benzo-6-diethylaminofluorane microcapsules were provided by Shenzhen Huancai Color Changing Technology Co., Ltd. and its components are melamine formaldehyde resin, methyl palmitate, ethyl stearate, 1,2-benzo-6-diethylaminofluorane, styrene maleic anhydride random copolymer. The waterborne acrylic resin was provided by Dulux Coatings Co., Ltd. Dulux waterborne acrylic resin coating consists of aqueous acrylic copolymer dispersion, matting agent, additive agent and water. Dulux Muyun Jingwei scratch resistant wood primer and finish were provided by Shanghai Dulux Co., Ltd. The main components include waterborne acrylic acid dispersion, additive agent, dulling agent, and the solid content is about 30.0%. Basswood substrates, width size 100 mm × 65 mm × 4 mm, had the uniform color after sanding pretreatment.

### 2.2. Preparation Method of UF @ Waterborne Acrylic Resin Microcapsules

The 27.0 g of 37% formaldehyde and 20.0 g urea were added to the beaker at a 1:1 molar ratio [34,35]. Then the 40 mL distilled water was added, and the mixture was placed in a magnetic stirrer for stirring. The triethanolamine was added dropwise, and the pH was adjusted to 8.0. Then the transparent UF prepolymer was obtained by stirring for 1.5 h at a water bath temperature of 70 °C and a stirring speed of 1200 rpm. Then the 1.37 g of sodium dodecylbenzene sulfonate white powder and 135.60 mL of distilled water were added to another beaker and stirred until completely dissolved. The sodium dodecylbenzene sulfonate solution with concentration of 1.0% was obtained as emulsifier. The 17.5 g of waterborne acrylic resin was emulsified. It was stirred in a water bath at 60 °C at 1200 rpm for 30 min. Then 1–2 drops of n-octanol were added for defoaming. Under stirring at 300 rpm, UF prepolymer was slowly added to the core material. The pH was adjusted with citric acid until the solution was completely dissolved. Finally, the polymer reacted slowly in a water bath for 3 h. The prepared microcapsules were filtered with ethanol and distilled water. The dried microcapsules were in powder form to facilitate the preparation of self-repairing waterborne coatings.

### 2.3. Preparation Method of Thermochromic Self-Repairing Bifunctional Coating

The orthogonal design of three factors and two levels were designed with “fluorane microcapsule content”, “aqueous acrylic resin microcapsule content” and “fluorane microcapsule addition method”, as shown in Table 2. According to the relevant report of Yan et al. [36], when the content of fluorane microcapsule in aqueous primer or finish is 15%, the thermochromic performance is better. Therefore, the horizontal range of “fluorane microcapsule content” is selected around 15.0%, so 10.0–20.0% is used to determine the best value. Thus, the content of microcapsules was determined to be 10.0%. Therefore, the horizontal range of “water-based acrylic resin microcapsule content” in this experiment was about 10.0%, that is, 5.0–15.0%.

The ingredients for the two microcapsules are shown in Table 3. The 1–4# samples are the materials corresponding to the orthogonal experiment. For example, 1# sample preparation: 0.2 g fluorane microcapsule was weighed and 1.8 g primer was mixed evenly. After coating the basswood substrate with SZQ four side film preparer (Chengdu Zhentong Trading Co., Ltd.), it was heated in the oven at 35 °C for 20 min, then the basswood was taken out. The surface was gently polished by 800# sandpaper and the floating powder removed [37,38]. The primer coating was completed and the coating process repeated twice. The coating method of the topcoat is the same as that of the primer. The preparation methods of other samples are the same as that of 1# samples. The prepared dry film thickness is about 60 μm.

### 2.4. Testing and Characterization

Test of coating rate of microcapsules: First, the 1.0 g microcapsule (*M*_1_) was weighed, and fully grinded. The amount of ethyl acetate was added to it, so that it could be thoroughly soaked in 72 h and replaced every 24 h. Then washed and filtered with deionized water. After drying, the quality of residual wall material (*M*_2_) was weighed. The coating rate (*C*) of microcapsules is calculated with the Formula (1).

*C* = (*M*_1_ − *M*_2_)/*M*_1_ ∗ 100% (1)

Self-repairing performance test of film: On the glass plate, manually scratch the coated water-based coating film with an art knife and immediately place it under the optical microscope. On a computer connected to an optical microscope, the width of the gap W_1_ (μm) was observed. After 5 days of standing at room temperature, the width of the gap W_2_ (μm) was measured again under the optical microscope, and the size of the gap was marked. In the later stage, the film gap width before and after scratching was compared to characterize whether the microcapsule had a certain self-repairing function to the film. The self-repair recovery rate is calculated with the Formula (2):

W = (W_1_ − W_2_)/W_1_ ∗ 100% (2)

Optical performance test: The test temperature range was set with the indoor temperature of 16 °C in Nanjing in winter as the starting point and the maximum indoor temperature of 40 °C in summer as the end point. The sample was heated by HGQ grating cutter (Guangzhou Keyu New Material Technology Co., Ltd., Guangzhou, China). At the same time, the temperature change in the coating surface was measured with a UT308H infrared handheld thermometer (Shenzhen Yimei Technology Co., Ltd., Shenzhen, China). The chromaticity parameters of the coating under 16–40 °C temperature rise and 40–16 °C cooling process were tested by SEGT-J portable chromatic aberration instrument (Shenyang Guotai precision test instrument Co., Ltd., Shenyang, China). L indicates lightness. The larger value shows that the color of the coated film is brighter, and the smaller value is darker. The “L”, “a” and “b” show the chromaticity of the coating film. The higher the value of L, the brighter the color of the coating; the larger the value of “a”, the redder the color of the film. The larger the b value, the color of the film tends to be yellow. The chromaticity parameters of the reversible color changing samples at 16 °C were taken as the reference points, and during two independent tests, the chromaticity values with various test temperatures were recorded. According to Hunter’s chromatic aberration equation, the chromatic aberration trend of samples at different temperatures was calculated. The chromatic aberration (ΔE) of the coating is calculated with the Formula (3).

ΔE = [(ΔL)^2^ + (Δa*)^2^ + (Δb*)^2^]^1/2^ (3)

Mechanical property test: In this experiment, a portable pencil hardness tester (Guangzhou biageda Precision Instrument Co., Ltd., Guangzhou, China) and 6H–6B pencil were used to measure the coating hardness. QFH-HG600 griddle knife film scriber (Tianjin Shengnuo Instrument Technology Co., Ltd., Tianjin, China) was selected to measure the film adhesion. The adhesive force is the best when the adhesion force is 0. QCJ-50 film impact tester (China Hebei yaoyang Instrument Equipment Co., Ltd., Hebei, China) was used to detect the shock resistance of the film. The impact strength is expressed in kg∙cm. The breaking elongation of the film was measured by a precision electronic machine (Suzhou jianzhuo Instrument Technology Co., Ltd., Suzhou, China). The tensile speed of the coating is 0.12 mm/min.

Microstructure test: The Olympus-CX23-Optical microscope (Jinan Taiyi Biotechnology Co., Ltd., Jinan, China) and Zeiss Evo10 scanning electron microscope (Beijing presys Instrument Co., Ltd., Beijing, China) were used to observe the structure of microcapsules and coatings. 

Infrared spectrum test: The chemical components of microcapsules and coatings were measured by Fourier infrared spectrometer (Germany Brooke Co., Ltd., Germany). 

All experimental errors were controlled within 5% and repeated 4 times, and the statistical significance of the experimental results was analyzed.

## 3. Results and Discussion

### 3.1. Properties and Morphology of UF @ Waterborne Acrylic Resin Microcapsules 

**Figure 1:**
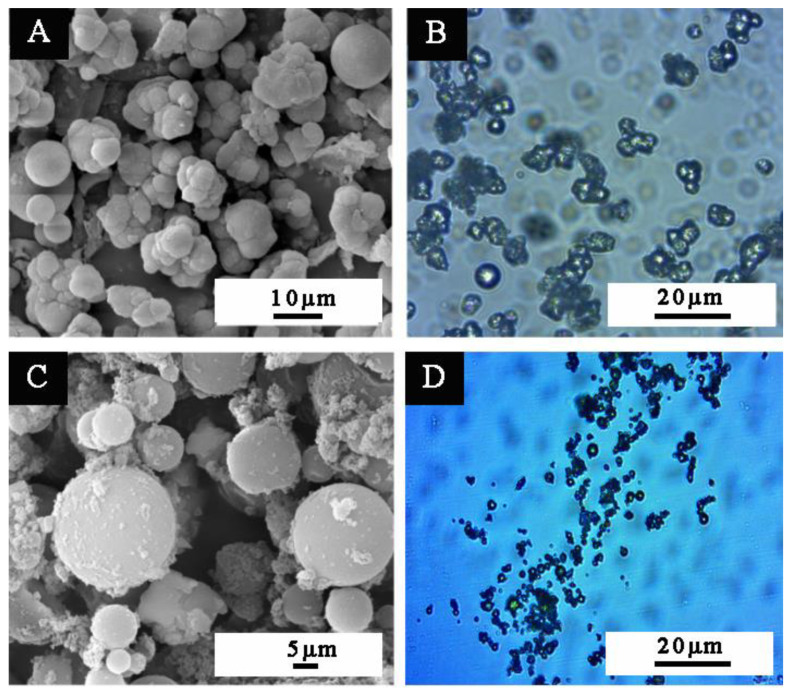
Morphology of waterborne acrylic resin microcapsules: SEM (**A**), OM (**B**) light diffraction ring phenomenon of waterborne acrylic resin microcapsules. Morphology of fluorane microcapsules: SEM (**C**), OM (**D**) light diffraction ring phenomenon of fluorane microcapsules.

**Figure 2:**
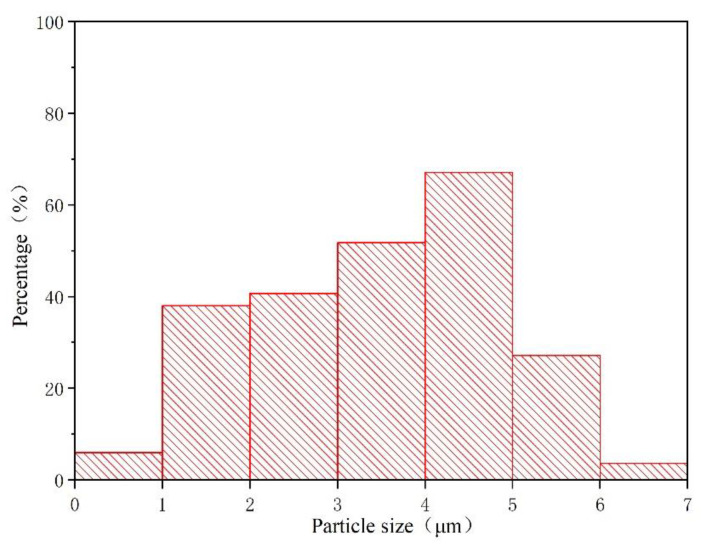
Particle size distribution of waterborne acrylic resin microcapsules.

According to the calculation of the coating rate, the coating rate of the prepared waterborne acrylic resin microcapsule is 57%. Figure 1 shows the micro morphology of waterborne acrylic resin microcapsules and fluorane microcapsules, respectively. Figure 1a shows the electron microscope of the waterborne acrylic resin microcapsule at high magnification. Figure 1c shows the scanning electron microscope of the fluorane microcapsule under high magnification. It can be seen from the diagram that the morphology of the two groups of microcapsules is round and granular, and some microcapsules are adhered together. As shown in Figure 2, the particle size of waterborne acrylic acid microcapsules is distributed evenly and the distribution is relatively narrow. The proportion of microcapsules with a particle size of 4–5 μm is the highest, which is the ideal microcapsule. The light produces a diffraction ring at the interface of the medium [39]. The diffraction pattern of the waterborne acrylic resin microcapsules and fluorane microcapsules shows that there are two different media, as shown in the OM diagrams (Figure 1b,d). The dark and transparent parts represent the wall material and core material, respectively. The microcapsules show a round shape and clear core wall structure.

**Figure 3:**
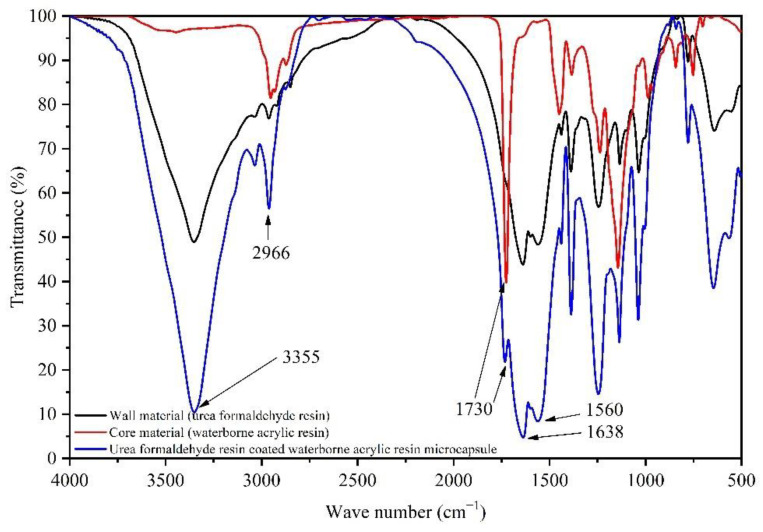
Infrared spectra of urea formaldehyde resin, waterborne acrylic resin and waterborne acrylic resin microcapsules.

Figure 3 shows the infrared spectrum of the wall material (UF), core material (waterborne acrylic resin) and UF @ waterborne acrylic resin microcapsules. The absorption peaks at 3355 cm^−1^ and 1560 cm^−1^ are N-H and C-N absorption peaks, respectively, which are the functional groups [40] of UF. The 1638 cm^−1^ belongs to the stretching vibration of C=O in UF. The characteristic peak of C-H is at 2966 cm^−1^. The 1730 cm^−1^ represents the absorption peak of C=O in aqueous acrylic resin [41]. The corresponding peaks also appeared in the infrared spectrum of the waterborne acrylic resin microcapsules. It was determined that the corresponding UF and waterborne acrylic resin were formed in the prepared microcapsules, and the components were not damaged. Therefore, the UF @ waterborne acrylic resin microcapsules were successfully prepared.

### 3.2. Orthogonal Experimental Analysis

**Figure 4:**
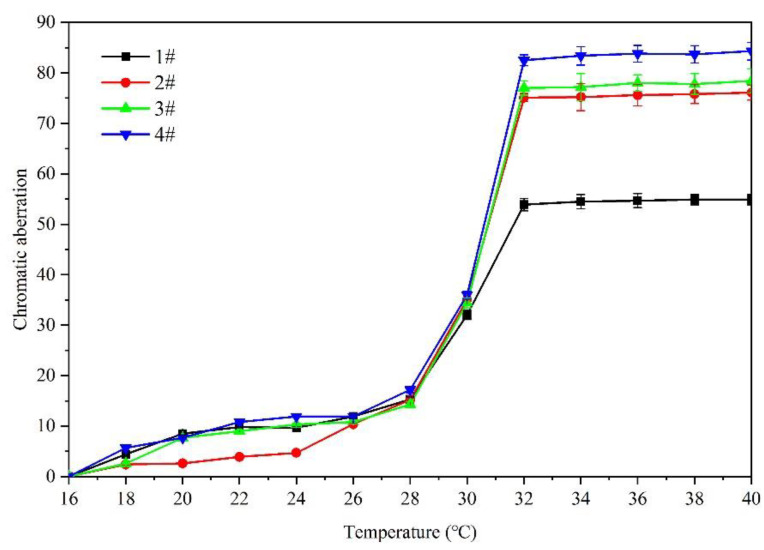
The temperature rise (16 °C to 40 °C) on color difference of film.

Table 4 shows the effect of the 16 °C to 40 °C temperature rise on the film colorimetric parameters of the microcapsule complex. The effect of temperature (16 °C to 40 °C) on the chromatic aberration of the coating film added with the microcapsule complex is shown in Figure 4. It is concluded that the color difference of coating 1–4# increases gradually with elevated temperature. Overall, the color difference of 1# is smaller than that of orthogonality 2–4#. The increase trend of the color difference of the coating film is small between 16 and 30 °C. The increase range of the color difference of the film becomes larger when the experimental temperature rises from 30 °C to 32 °C, and it tends to a maximum at 32 °C. The chromatic aberration does not change significantly and is in a stable state when the temperature is within the range of 32–40 °C. It can be shown that the discoloration temperature range of the sample is 30–32 °C, and thermochromism occurs at 32 °C.

**Figure 5:**
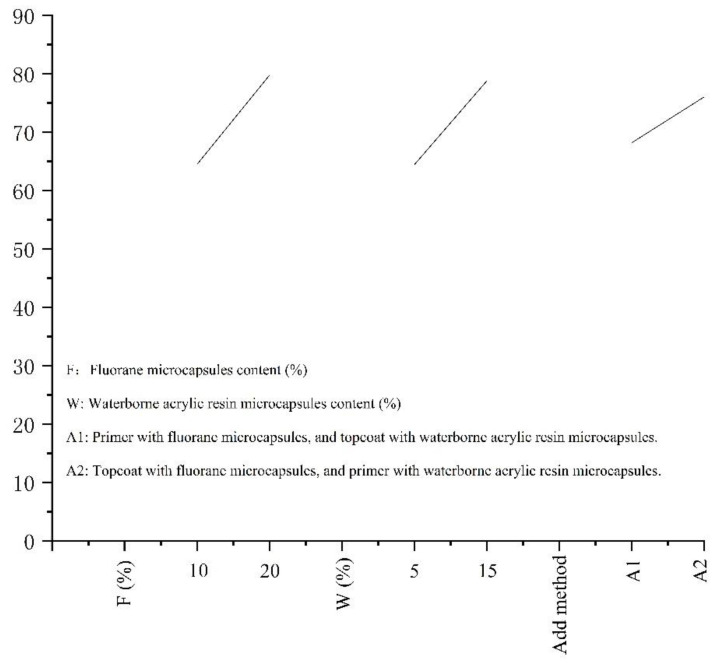
Results of orthogonal experiment.

In order to optimize the thermochromic performance of the film, the color difference of 16–32 °C was used as the experimental result to analyze the orthogonal experiment in Table 5 and Figure 5. The results show that the main factor leading to the chromatic aberration of the film is the “fluorane microcapsule content”. The second is the “content of waterborne acrylic resin microcapsules” and “addition method of fluorane microcapsules”. According to the mean 1 and mean 2, it is determined that the amount of waterborne acrylic resin microcapsules is 15%. The fluorane microcapsules were added into the topcoat, while the waterborne acrylic resin microcapsules were added into the primer. It has a greater influence on the color difference of the coating. Therefore, the next step is to change the content of the fluorane microcapsules and analyze the effects of the “fluorane microcapsule content” (5.0%, 10.0%, 15.0%, 20.0%, 25.0%, 30.0%) on various properties of the coating.

### 3.3. Single Factor Experimental Results and Analysis of “Fluorane Microcapsule Content”

#### 3.3.1. Effect of Fluorane Microcapsule Content on Optical Properties of Coating 

**Figure 6:**
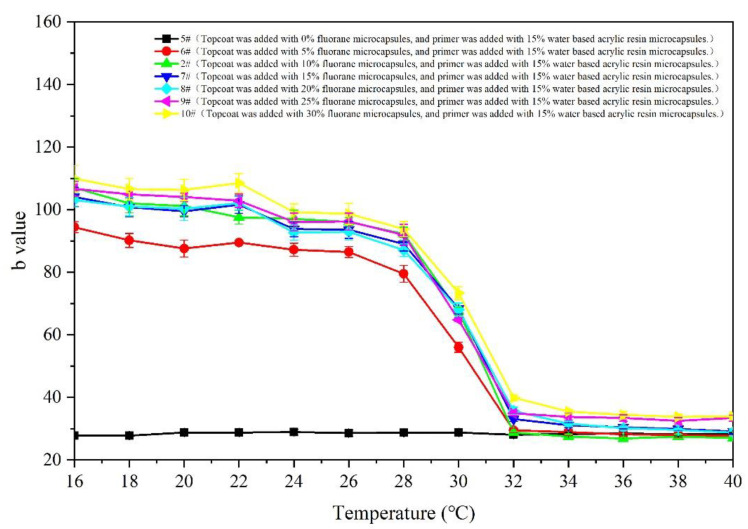
The temperature rise (16 °C to 40 °C) on b value of film.

Figure 6 shows the chromaticity parameters [42] of 5–10# samples with different contents of fluoranthene microcapsules. It is known that for the whole trend, with the increase in test temperature, the b value of the chromaticity parameters of 5# (excluding fluoranthene microcapsule coating) is maintained at 27.0–29.0, almost unchanged [43]. Figure 5 shows the decreasing b value of the fluorane coating with 5.0–30.0% content. It indicates that the film changes from yellow to colorless. For the whole trend, the b value of each coating in the 16–28 °C interval does not change significantly. When the test temperature is 30 °C, the b value of the film begins to show a downward trend. The b value of the coating tends to be stable between 32 °C and 40 °C. Therefore, it can be preliminarily determined that the film has changed to being colorless at the node of 32 °C.

**Figure 7:**
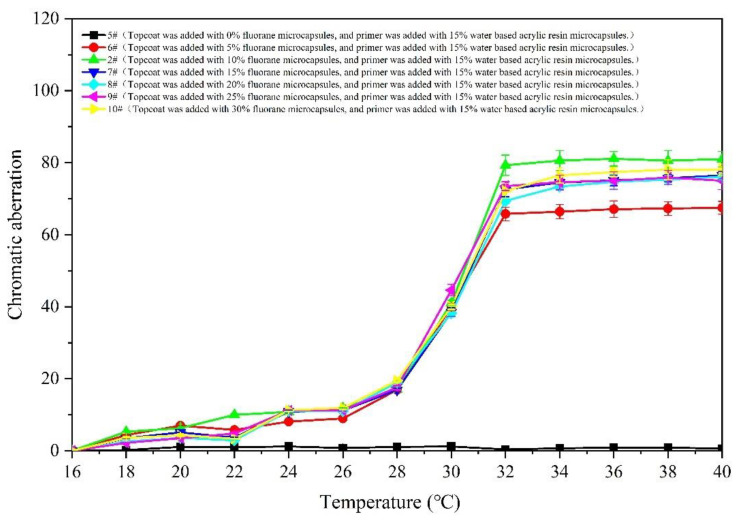
The influence of temperature rise on color difference of coating.

**Figure 8:**
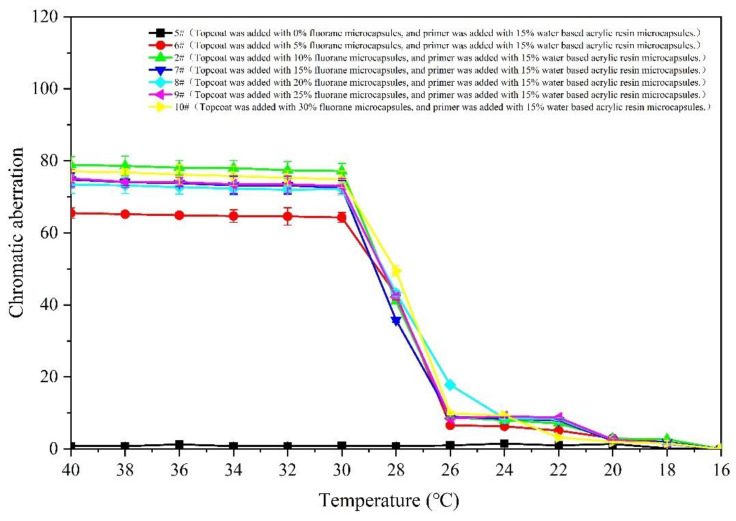
The influence of cooling on color difference of coating.

Figure 7 and Figure 8 show the changes in the chromatic aberration of 5–10# samples (with different accounts of fluorane microcapsules, primers and 15% waterborne acrylic resin microcapsules). The color difference increment of all samples in the 16–28 °C interval is very small, almost all are less than 20 °C. When the film continues to heat up to 30 °C, the increase range of the color difference becomes larger. The color difference reaches the maximum when the temperature is 32 °C. In combination with Figure 5, it can be preliminarily determined that the color of the film has changed at the node of 32 °C. Among the samples, for 6# (the coating film with 5.0% fluorane microcapsules added to the topcoat and 15.0% waterborne acrylic resin microcapsules added to the primer), the color difference value at 32 °C is smaller than that of other coatings. The difference in the chromatic aberration between 2# and 7–10# at 32 °C is not significant, 7# is at the maximum value of 79.3 ± 2.8, followed by 7# at 72.6 ± 2.0. The color values of the primers and topcoat were, respectively, added with the fluorane and waterborne acrylic resin microcapsules. The change trend of the temperature was consistent with that of the fluorane microcapsules. It is indicated that this way of adding will not change the temperature range of the chromatic aberration, and the film prepared can still achieve temperature-reversible color change.

**Figure 9:**
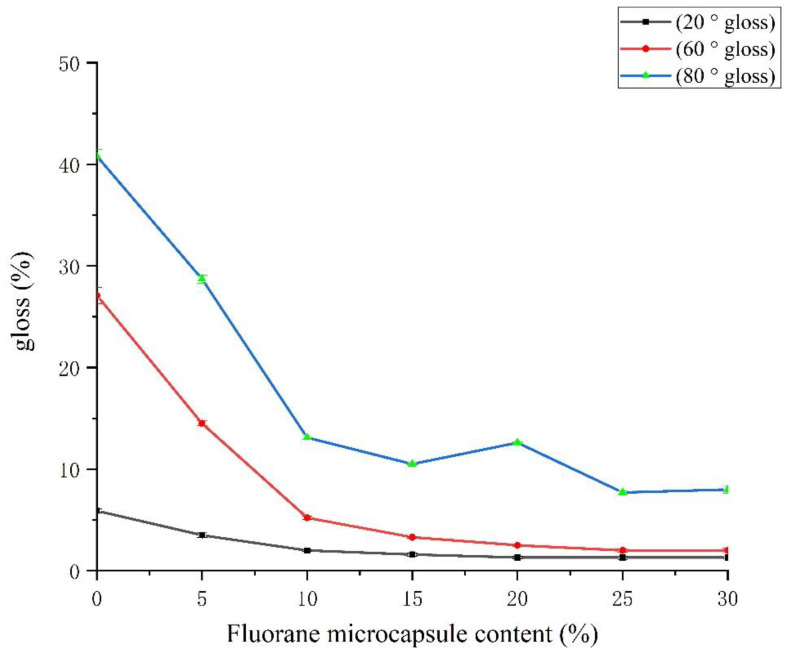
The fluorane microcapsule content on film gloss.

The decreasing trend of the film gloss with the additional amount of fluorane microcapsules is shown in Table 6 and Figure 9. The gloss of 5# coating is higher than that of 6–11# coatings with different contents of fluorane microcapsules added to other topcoats. This may be due to the fact that 5# topcoat does not contain fluorane microcapsules. The primer is added with 15% waterborne acrylic resin microcapsules, and the surface of the topcoat is smoother, so the gloss is relatively high. From the overall trend, the gloss of the film decreases gradually with the increase in fluorane microcapsule content in the topcoat. The reason may be that the roughness of the coating surface increases with the increase in fluorane microcapsule content in the topcoat. This leads to light scattering, which reduces the gloss of the coated surface [44].

#### 3.3.2. Effect of Fluorane Microcapsule Content on Mechanical Properties of Coating

**Figure 10:**
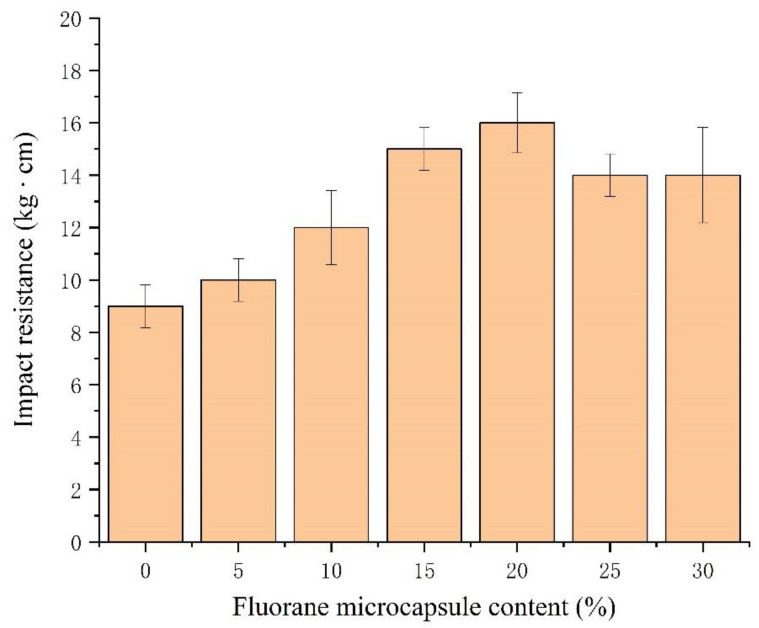
The fluorane microcapsule content on film impact resistance.

**Figure 11:**
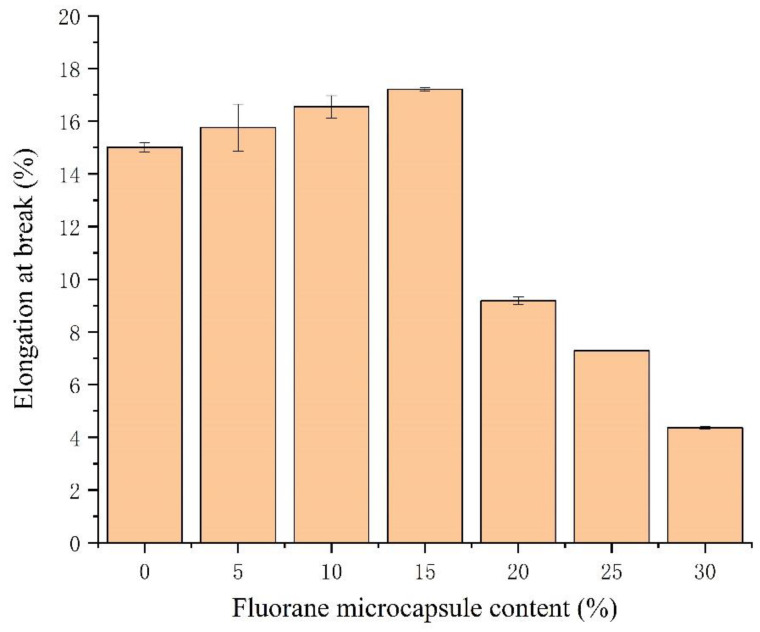
The fluorane microcapsule content on film elongation at break.

The effect of the fluorane microcapsule content on film hardness, impact resistance, adhesion and elongation at break is shown Figure 10 and Figure 11. The experimental results showed that the hardness (Table 7), impact resistance and elongation at break increased first and then decreased with the increase in the content of fluorane. The adhesion decreased from 0 to 1. When the content of the fluorane microcapsules is 0–15.0%, the adhesion of the film is good and the grade is 0. This indicates that the proper addition of microcapsules to the original coating film can ensure excellent adhesion. The adhesion drops to grade 1 when the content exceeds 15.0%. The adhesion drops to grade 1 when the content exceeds 15.0%. This shows that excessive additional content will lead to the decrease in the bonding force of the mechanical glue nail between the wood and the film, resulting in the decrease in the adhesion [45]. When the content of the fluorane microcapsules is 15.0%, the hardness is 4H, the adhesion force is 0, the impact resistance is 15.0 ± 0.8 kg∙cm and the elongation at break is 17.2%. The results show that when the content of the microcapsules in the coating reaches a certain level, the microcapsules are evenly distributed in the coating matrix. The microcapsule particles have good compatibility with the coating. Therefore, it has good impact resistance. The wall material of the microcapsule has good compressive strength and toughness when the coating is impacted. To some extent, it can play a buffer role, thus reducing the film of the impact resistance [46,47]. When the film is stretched by external force, the wall material UF in the primer waterborne acrylic resin microcapsule and the core material released by stretching will be repaired in time. Therefore, the addition of certain microcapsules will increase the toughness of the film and increase the elongation at break. However, as the amount of addition increases, the microcapsules form agglomeration on the surface of the film. This reduces the film’s flexibility [48].

### 3.4. Microstructure and Infrared Spectrum Analysis

**Figure 12:**
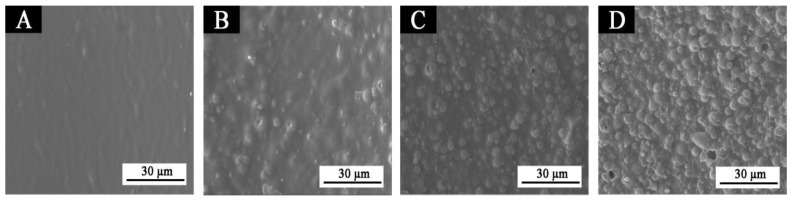
SEM of films with different concentrations of fluorane microcapsules: (**A**) 5#, (**B**) 2#, (**C**) 8#, (**D**) 10#.

SEM images of fluorane microcapsules with different contents added to the topcoat are shown in Figure 12. Figure 12a–d shows the water-based coating film with different amounts of fluorane microcapsules added to the finish paint. With the continuous addition of fluorane microcapsules in the topcoat, the morphology of the particles on the coating surface tends to be obvious. When the content is 30.0%, there are small holes on the surface of the film. This may be due to too much addition of microcapsules, resulting in a small number of bubbles being generated during drying, or, in the grinding stage, due to the excessive force of sandpaper, the paint film will fall off or scratch slightly, and the surface will be not smooth in the electron microscope [49,50]. Therefore, it is very important to control the addition of microcapsules.

**Figure 13:**
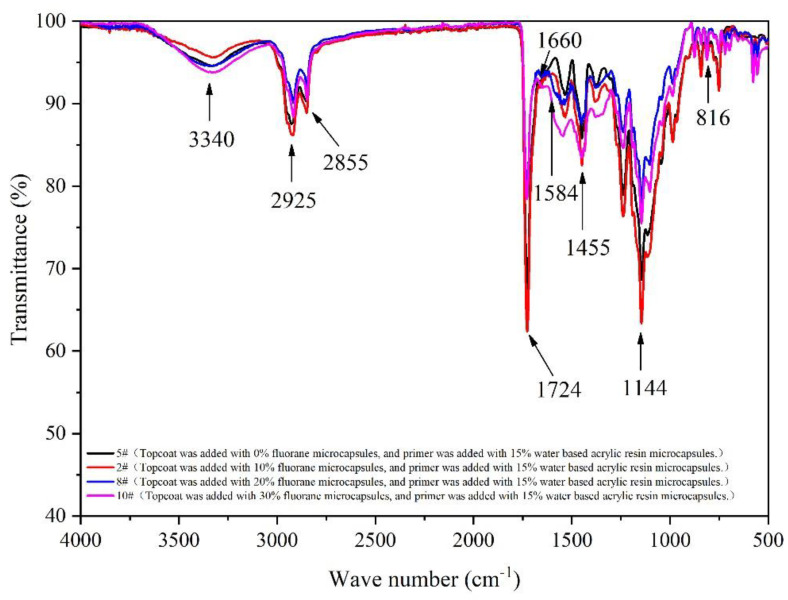
Infrared spectra of coating films with different contents of fluorane microcapsules.

The coatings with different amounts of fluoranthene microcapsules are shown in Figure 13 infrared spectrogram. At 3340 cm^−1^, there are tensile vibration peaks of -NH and -OH. The 2925 cm^−1^ peak is the tensile vibration of -CH_3_. The 1144 cm^−1^ is known to be the telescopic vibration absorption of C-O-C. The 1584 cm^−1^ and 816 cm^−1^ points are the absorption peaks and flexural vibration absorption peaks of the three zine rings. The 1660 cm^−1^ peak is the expansion vibration of C=O in the UF @ waterborne acrylic resin microcapsules’ wall material. The 1455 cm^−1^ value is the characteristic peak of C-H. The 1730 cm^−1^ peak is accompanied by strong carbonyl characteristic absorption. It not only represents the characteristic peak of C=O in the core of the fluorane microcapsule, but also represents the characteristic peak of C=O in 1,2-benzo-6-diethylaminofluorane in the core material of the fluorane microcapsule in the topcoat. When the fluorane microcapsules are added into the paint, no peaks disappear or appear with the change in fluorane microcapsule content. It shows that there is no difference in the composition of the coating film with the different content of fluorane microcapsules. This shows that there is no reaction between the two microcapsules with aqueous coating on the surface of the basswood. Different contents of fluorane microcapsules were added into the topcoat and 15.0% water-based acrylic resin microcapsules in the primer can still have a thermochromic effect.

### 3.5. Self-Repairing Property

**Figure 14:**
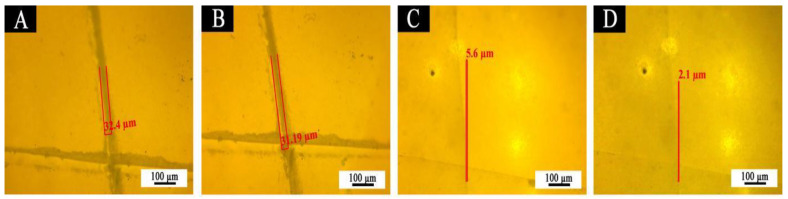
OM images of film: before healed (**A**) 11#, (**C**) 7#, after healed (**B**) 11#, (**D**) 7#.

Figure 14 is a contrast map of 11# and 7# before and after restoration under an optical microscope. Figure 14a is an optical microscope view of 11# sample immediately after the coating film is scratched by the blade. Figure 14b is an optical microscope view after standing for 5 days. Figure 14c shows the 7# sample after the water-based acrylic resin microcapsule is added. Figure 14d is an optical microscope view after standing for 5 days. The gap width before and after coating repair is used to characterize whether the coating has a repair function. When the water-based acrylic resin microcapsules were added, the crack width of the 7# coating film reduced after 5 days. The width of the crack was 5.6 μm before the 7# coating was repaired. After 5 days at room temperature, the width of the crack observed again was 2.1 μm. The gap width of the coating film was significantly reduced by 3.5 μm. The repair rate reached 62.5%. The 11# coating film without aqueous acrylic resin microcapsules is shown in Figure 14a,b. The crack width of the coating film after 5 days reduced by 1.2 μm. It had little change. This shows that the cracks in the film without microcapsules were hardly repaired. As a core material, the water-based acrylic resin is a one component repair agent without a curing agent and catalyst. The self-healing microcapsules were prepared by two-step in situ polymerization to inhibit the generation and expansion of microcracks in the coating, which is suitable for the repairing of waterborne coatings.

The self-repairing rate of the paint film prepared by the independent addition method of “the fluorane and lac resin microcapsules” is only 12.3% [36]. In comparison, the two groups adopt the method of independent addition, which shows that the coating method can enable the paint film to self repair, but the repair effect of the waterborne acrylic resin is better than that of the lac resin, which has a better function for inhibiting cracks.

Compared with the mixed addition method [51], the best result is to add two kinds of microcapsules into the primer at the same time, and the repair rate is 58.4%. The independent addition method in this article is to add two kinds of microcapsules in the topcoat and primer, respectively, and the repair rate is 62.5%, which is better than the mixed addition method. The reason may be that when the coating film produces microcracks, the capsule wall of the water-based acrylic resin microcapsule in the primer breaks due to mechanical stress. At this time, the waterborne acrylic resin is released and can respond quickly. In the mixed addition, there are two kinds of microcapsule polymers. Because the content of the fluorane microcapsules is higher than that of the waterborne acrylic microcapsules, the efficiency and effect of the repair will be affected to some extent. This shows that the film with “15.0% fluorane microcapsules added to the topcoat and 15.0% waterborne acrylic resin microcapsules added to the primer” can achieve a certain degree of self repair. 

## 4. Conclusions

The encapsulation efficiency of the waterborne acrylic resin microcapsules was 57%. The infrared spectrum proved that the UF @ waterborne acrylic resin microcapsules were prepared successfully. Three factors were selected by orthogonal experiment, and the results of the “fluorane microcapsule content, water-based acrylic resin microcapsule content and addition method” were not significant. The content of the fluorane microcapsules obtained by the range method is the most influential factor. It was concluded that the comprehensive performance was better when the amount of fluorane microcapsule in the film was 15.0%. The results of the optical, mechanical, microstructure and self-repairing experiments show that the color difference at 32 °C is 72.6 ± 2.0, the gloss at 60° is 3.3%, the hardness is 4 H, the impact resistance is 15.0 ± 0.8 kg∙cm, the adhesion is grade 0 and the elongation at break is 17.2%. The repair rate reached 62.5%. Comparing the two methods of independent addition and mixed addition, the repair effect of the waterborne acrylic resin is the best, and the independent addition can coordinate the two microcapsules to achieve a better self-healing effect. In general, the coating film of the “15.0% fluorane microcapsule added to the topcoat and 15.0% waterborne acrylic resin microcapsule added to the primer” has the dual effects of a rapid response to thermochromism and the inhibition of microcracks. However, the urea formaldehyde resin selected in this paper contains formaldehyde, and the release of formaldehyde and its impact on the environment have not been tested. The waterborne acrylic acid microcapsules have agglomeration phenomenon, and the coating rate needs to be improved. In general, the thermochromic self healing water-based coating significantly broadens the application range.

## Figures and Tables

**Table 1:** Materials and reagents.

Experimental Materials	Purity	Manufacturer
37.0% formaldehyde	analytically pure	Nanjing Chemical Reagent Co., Ltd., Nanjing, China
urea	analytically pure	Nanjing Chemical Reagent Co., Ltd., Nanjing, China
triethanolamine	analytically pure	Nanjing Chemical Reagent Co., Ltd., Nanjing, China
sodium dodecyl benzene sulfonate	analytically pure	Tianjin Beichen Fangzheng reagent factory, Tianjin, China
n-octanol	analytically pure	Sinopharm Chemical Reagent Co., Ltd., Shanghai, China
citric acid monohydrate	analytically pure	Sinopharm Chemical Reagent Co., Ltd., Shanghai, China
absolute ethanol	analytically pure	Suzhou Jiading Chemical Technology Co., Ltd., Suzhou, China
ethyl acetate	analytically pure	Hubei handafei Biotechnology Co., Ltd., Hubei, China
waterborne acrylic acid	-	Shanghai Dulux Co., Ltd., Shanghai, China

**Table 2:** The orthogonal experimental test of discoloration and self-repairing film.

Sample (#)	Content of Fluorane Microcapsule (%)	Content of Waterborne Acrylic Resin Microcapsules (%)	Addition Method of Fluorane Microcapsule
1#	10.0	5.0	Primer with fluorane microcapsules, and topcoat with waterborne acrylic resin microcapsules.
2#	10.0	15.0	Topcoat with fluorane microcapsules, and primer with waterborne acrylic resin microcapsules.
3#	20.0	5.0	Topcoat with fluorane microcapsules, and primer with waterborne acrylic resin microcapsules
4#	20.0	15.0	Primer with fluorane microcapsules, and topcoat with waterborne acrylic resin microcapsules

**Table 3:** Ingredients of thermochromic self-repairing coatings.

Sample (#)	Fluorane Microcapsules (%)	Waterborne Acrylic Resin Microcapsule (%)	Fluorane Microcapsules (g)	Waterborne Acrylic Resin Microcapsule (g)	Primer (g)	Topcoat (g)	Thermochromic Self-Repairing Coating (g)
1#	10.0	5.0	0.2	0.1	1.8	1.9	4.0
2#	10.0	15.0	0.2	0.3	1.7	1.8	4.0
3#	20.0	5.0	0.4	0.1	1.9	1.6	4.0
4#	20.0	15.0	1.4	0.3	1.6	1.7	4.0
5#	0	15.0	0	0.3	1.7	2.0	4.0
6#	5.0	15.0	0.1	0.3	1.7	1.9	4.0
7#	15.0	15.0	0.3	0.3	1.7	1.7	4.0
8#	20.0	15.0	0.4	0.3	1.7	1.6	4.0
9#	25.0	15.0	0.5	0.3	1.7	1.5	4.0
10#	30.0	15.0	0.6	0.3	1.7	1.4	4.0
11#	15.0	0	0.3	0	2.0	1.7	4.0

**Table 4:** The temperature rise (16 °C to 40 °C) on colorimetric aberration of film.

Sample (#)	Chromatic Aberration	16 °C	18 °C	20 °C	22 °C	24 °C	26 °C	28 °C	30 °C	32 °C	34 °C	36 °C	38 °C	40 °C
1	ΔE	0	4.4 ± 0.1	8.5 ± 0.2	9.8 ± 0.1	9.7 ± 0.2	11.9 ± 0.2	15.3 ± 0.4	32.0 ± 0.8	53.9 ± 1.2	54.5 ±1.4	54.7 ± 1.4	54.9 ± 1.0	54.9 ± 1.0
2		0	2.4 ± 0.1	2.6 ± 0.1	3.9 ± 0.1	4.7 ± 0.1	10.4 ± 0.2	15.2 ± 0.4	35.1 ± 0.1	75.1 ± 0.8	75.2 ± 2.7	75.6 ± 2.1	75.8 ± 1.8	76.1 ± 1.5
3		0	2.6 ± 0.1	7.7 ± 0.2	9.0 ± 0.2	10.3 ± 0.2	10.8 ± 0.4	14.3 ± 0.5	34.4 ± 0.9	77.0 ± 1.4	77.2 ± 2.7	78.0 ± 1.6	77.8 ± 2.0	78.4 ± 2.4
4		0	5.7 ± 0.1	7.7 ± 0.2	10.8 ± 0.2	11.9 ± 0.1	11.9 ± 0.1	17.2 ± 0.5	36.0 ± 0.8	82.5 ± 1.0	83.4 ± 1.8	83.8 ± 1.6	83.7 ± 1.7	84.3 ± 1.7

**Table 5:** Results of orthogonal experiment.

Sample	Fluorane Microcapsule Content (%)	Content of Waterborne Acrylic resin Microcapsules (%)	Add Method	Temperature of 16–32 °C Color Difference Results
1#	10.0	5.0	Primer with fluorane microcapsules, and topcoat with waterborne acrylic resin microcapsules.	53.9 ± 1.2
2#	10.0	15.0	Topcoat with fluorane microcapsules, and primer with waterborne acrylic resin microcapsules.	75.1 ± 0.8
3#	20.0	5.0	Topcoat with fluorane microcapsules, and primer with waterborne acrylic resin microcapsules.	77.0 ± 1.4
4#	20.0	15.0	Primer with fluorane microcapsules, and topcoat with waterborne acrylic resin microcapsules.	82.5 ± 1.0
Mean 1	64.500	64.450	68.200	
Mean 2	79.750	78.800	76.050	
Range	15.250	13.350	7.850	
Significance	-	-	-	

**Table 6:** The fluorane microcapsule content on film gloss.

Sample (#)	Fluorane Microcapsule Content (%)	20 ° Gloss (%)	60 ° Gloss (%)	85 ° Gloss (%)
5#	0	5.9 ± 0.2	27.1 ± 0.8	40.8 ± 0.6
6#	5.0	3.5 ± 0.2	14.5 ± 0.2	28.7 ± 0.3
2#	10.0	2.0	5.2 ± 0.1	13.1 ± 0.1
7#	15.0	1.6 ± 0.1	3.3	10.5
8#	20.0	1.3 ± 0.1	2.5	12.6
9#	25.0	1.3 ± 0.1	2.0 ± 0.1	7.7 ± 0.1
10#	30.0	1.3	2.0 ± 0.2	8.0 ± 0.3

**Table 7:** The fluorane microcapsule content on mechanical properties.

Sample (#)	Fluorane Microcapsule Content (%)	Hardness (H)	Adhesion (grade)	Impact Resistance (kg∙cm)	Elongation at Break (%)
5#	0	2	0	9.0 ± 0.8	15.0 ± 0.1
6#	5.0	3	0	10.0 ± 0.8	15.7 ± 0.8
2#	10.0	3	0	12.0 ± 1.4	16.5 ± 0.4
7#	15.0	4	0	15.0 ± 0.8	17.2
8#	20.0	5	1	16.0 ± 1.2	9.1 ± 0.1
9#	25.0	4	1	14.0 ± 0.8	7.2
10#	30.0	4	1	14.0 ± 1.8	4.3

## Data Availability

Not applicable.
